# Feasibility and acceptability of patient partnership to improve access to primary care for the physical health of patients with severe mental illnesses: an interactive guide

**DOI:** 10.1186/s12939-015-0200-0

**Published:** 2015-09-14

**Authors:** Jean-François Pelletier, Alain Lesage, Christine Boisvert, Frédéric Denis, Jean-Pierre Bonin, Steve Kisely

**Affiliations:** Department of Psychiatry, University of Montreal, Centre de recherche de l’Institut universitaire en santé mentale de Montréal, 7401, Hochelaga Street, Montreal, QC H1N 3M5 Canada; Department of Psychiatry, Yale School of Medicine, New Haven, USA; International Program for Participatory Action Research, Montréal, Canada; La Chartreuse Psychiatric Centre, Dijon, France; University of Franche-Comte, Besançon, France; Faculty of Nursing, University of Montreal, Montreal, Canada; School of Medicine, The University of Queensland, Brisbane, Australia

**Keywords:** Strategy for patient-oriented research, Interactivity, User-led research, Global health, Personalization, Participation, Small group learning, Patient partnership

## Abstract

**Introduction:**

Even in countries with universal healthcare systems, excess mortality rates due to physical chronic diseases in patients also suffering from serious mental illness like schizophrenia is such that their life expectancy could be lessened by up to 20 years. The possible explanations for this disparity include: unhealthy habits (i.e. smoking; lack of exercise); side-effects of psychotropic medication; delays in the detection or initial presentation leading to a more advanced disease at diagnosis; and inequity of access to services. The main objective of this paper is to explore the feasibility and acceptability of patient partnership for developing an interactive guide to improve access to primary care providers for chronic diseases management and health promotion among patients with severe mental illnesses.

**Methods:**

A participatory action research design was used to engage patients with mental illness as full research partners for a strategy for patient-oriented research in primary care for persons with schizophrenia who also have chronic physical illnesses. This strategy was also developed in partnership with a health and social services centre responsible for the health of the population of a territory with about 100,000 inhabitants in East-end Montreal, Canada. A new interactive guide was developed by patient research partners and used by 146 participating patients with serious mental illness who live on this territory, for them to be better prepared for their medical appointment with a General Practitioner by becoming more aware of their own physical condition.

**Results:**

Patient research partners produced a series of 33 short videos depicting signs and symptoms of common chronic diseases and risk factors for the leading causes of mortality and study participants were able to complete the corresponding 33-item questionnaire on an electronic touch screen tablet. What proved to be most relevant in terms of interactivity was the dynamic that has developed among the study participants during the small group learning sessions, a training technique designed for healthcare professionals that was adapted for this project for, and with patient partners.

**Conclusion:**

This research has shown the feasibility and acceptability of patient partnership and patient-oriented research approaches to the R&D process of a new medical tool and intervention for patients with serious mental illness, and its acceptability for addressing inequity of this disadvantaged population in terms of access to primary care providers.

## Introduction

Chronic diseases are the leading causes of death in Canada with more than 75 % of deaths attributable to one of the following disorders: cancer, cardiovascular disease, diabetes, kidney disease and respiratory disease. While the causes of chronic illnesses are not fully known, research has identified a range of apparently interrelated personal, social, economic, and environmental factors associated with the onset of those illnesses. Mortality from physical illnesses is over 70 % higher in psychiatric patients in relation to that of the general population, even after adjusting for demographics, including socio-economic status. Thus, people with schizophrenia have a substantially raised risk of premature death, and of great concern is that this risk might be increasing, rather than decreasing [1]. Although many factors are likely to contribute to the poor health of this population, explanations usually point to lifestyle factors (e.g. lack of exercise, alcohol and tobacco use). However, mortality remains high for this population even after adjusting for behavioural risk factors such as smoking, physical activity and body mass index. Patients with mental illness are more likely to delay seeking care, which is likely a contributor to the increase in mortality among these patients. It has also been shown that people with mental illness have inequitably less access to primary care providers, partly due to lack of thorough investigation and poor communication skills [2, 3].

All mental disorders are associated with increased risk of premature death [4]. Excess mortality rates due to physical chronic diseases in patients also suffering from serious mental illness like schizophrenia are such that their life expectancy could be lessened by up to 20 years [5–7]. The possible explanations for this disparity include: unhealthy habits (e.g. smoking, lack of exercise), side-effects of psychotropic medication; delays in the detection or initial presentation leading to a more advanced disease at diagnosis, and iniquity in the access to services in terms of care trajectories [8]. In effect, despite showing no greater incidence of chronic diseases, people with a mental illness, especially severe mental illness (SMI: schizophrenia, schizotypal disorder and delusional disorder, as defined in Chapter 5 of the International Classification of Diseases) are clearly less likely to receive or to adhere to adequate treatment [9]; this is in spite of consultation rates being generally high among this population. The main causes of mortality in patients with schizophrenia are the same as for the rest of the population (cardiovascular diseases and cancer), but individuals with SMI are prone to many different physical health problems. And while these diseases are also prevalent in the general population, their impact on individuals with SMI is significantly increased [10]. Yet, there is a greater delay for vital medical and surgical interventions for these patients compared to the general population [11].

To address this important public health problem, we are currently implementing a strategy of patient-oriented research for people with schizophrenia who are attending primary care for treatment of comorbid physical illness in the East-end of Montreal, Canada. Among the objectives is the development of a new Interactive Guide for Medical Appointments (IGMA). This paper reports on the contribution of people with mental illness to the research and development process (R&D) of the IGMA. We are particularly interested in the concept of diagnostic overshadowing; the over attribution of symptoms to any underlying or chronic condition, resulting in missed diagnoses and the improper management of conditions [2]. This is especially problematic in people with mental illness because they have higher rates of morbidity and shorter lifespans than the general population and evidence shows that enhancement of primary care services for such disadvantaged populations is essential to reducing health and health care inequities [12].

## Materials and methods

This study was reviewed and approved by the Institutional Review Board of *Institut universitaire en santé mentale de Montréal*, in the Canadian province of Quebec. It is being piloted by mental health service users who interact with study participants in small group learning sessions to document their perception of their own physical condition through the use of the IGMA. The IGMA displays self-administered questions targeting the signs and symptoms of common chronic diseases and risk factors for the leading causes of mortality. It was developed to assist in the diagnosis of physical illnesses in people with SMI because SMI can impede the ability of medical professionals to diagnose and address ongoing physical illnesses. The IGMA survey can therefore be used by medical professionals to ensure timely diagnosis of physical issues among patients with mental illnesses, and can also be used by patients to become more aware of their own physical condition and to feel more comfortable in engaging themselves in health promotion activities. It can also provide social support when used and discussed in small groups.

Small group learning is an educational approach that allows participants to develop problem solving, interpersonal, presentational and communication skills that are difficult to develop in isolation, and require feedback and interaction with other individuals. The goals of small group learning include the following: 1) to reinforce knowledge through problem solving; 2) plumb the depths of a problem; 3) test assumptions; 4) generate hypotheses and practice critical reasoning; 5) collaborate with peers, and; 6) to receive feedback. This approach has been shown to be particularly effective for improving doctor-patient communication [13]. For this study we presume that, on the other way around, it can also improve patient-doctor communication.

Difficult communication, social distance and the overall poor quality of interactions between health care providers and persons with a lower socioeconomic status, which is the case with most patients with SMI, have been identified as a barrier to healthcare for disadvantaged populations in Quebec [14] and elsewhere [15]. Therefore, the IGMA has been developed to facilitate this patient-doctor communication through a participatory action research type of equitable participation that allows non-academic members of the co-research team to immediately benefit from the research findings and to become directly involved in the knowledge translation process [10, 16].

### Participatory development process and governance

Participatory action research (PAR) is an approach that involves study participants in all aspects of the process, from conceptualization to data collection, through interpretation and dissemination of findings. Involving study participants as partners is a fundamental tenant of the PAR approach [17]. Co-learning, building on strengths and acknowledgement of privilege and power are additional characteristics. This project is participatory in many ways, and based on the field notes and on researchers’ reflective notes, this paper reports on the contribution of patient research partners in the R&D of the IGMA as it relates to access and equity.

The research team followed as strictly as possible the development process that was implemented in Australia by the Queensland Government with the *Activate: Mind & Body* project [18]. At the time of the study, very significant structural reforms were underway in Quebec. With the enactment of Law 10 the Quebec government tabled major overhaul of the health and social services network in order to modify its organization and governance, in particular by abolishing the regional agencies and reducing the number of administrative levels, the number of officers and boards of directors. As of April 1st 2015, the province’s 18 health and social services regional agencies and 182 health institutions have been amalgamated into just 28 institutions for the stated purpose of optimizing network efficiency. To ensure project acceptability in the eyes of practitioners in the field, it was important to underline its bottom-up rather than top-down nature. The patient partner expertise has been put at the forefront as a branding for the project, so that this project is an example of a patient-centered and even patient-led approach rather than centered on institutions’ needs. Thus, this project aimed to decisively improve the physical and oral health outcomes of those Australians with SMI by working collaboratively with public mental health services, primary health care professionals and non-government organisations to provide support and education to consumers of mental health services and their carers. With the IGMA project, patients are also actively involved in such a process as research partners throughout (patient research partners). For instance, this project was coordinated by patients who are members of the International Program for Participatory-Action Research (IPPAR), a non-profit organisation which is based at *Institut universitaire en santé mentale de Montréal* (IUSMM: Montreal Mental Health University Institute). The IPPAR is a consumer- and carer-run agency that specializes in participatory research for citizenship-oriented mental health policies and systems transformation [19]. The IPPAR is also a 5 year research program funded by the Canadian Institutes of Health Research (knowledge translation priority announcement) and the *Fonds de la recherche du Québec – Santé*. The mission of the IPPAR is twofold:

- to translate the experiential knowledge of consumers and carers into scientific evidence;
- to experiment the research and scientific milieu as an inclusive workplace for them.

The development of the IGMA has been a collaborative initiative based on expert knowledge and practice with multiple resources and stakeholders, and patients were involved in the R&D process from inception on. First, 2 patients were members of an Advisory Board that provided leadership and advice to the Co-Research Team for the adaptation of the Australian *Activate Mind and Body* guidelines to Montreal. The Advisory Board and Co-Research Team consisted of expert practitioners from a range of fields, with some of them being involved in both, while the IPPAR was solely comprised of service users and carers (Table 1).

**Table 1:** Composition of the Advisory Board, the Co-Research Team and the IPPAR

Expert Practitioners	Advisory Board	Co-Research Team	IPPAR
Physician with mental illness*	✓	✓	✓
Psychiatrist*	✓	✓	
Director of the IPPAR*	✓	✓	✓
Investigator familiar with the Australian Guidelines*	✓		
Adjunct director of the local health authority	✓	✓	
General Practitioner	✓	✓	
Practicing nurse	✓		
Family member (mother of a person with SMI)	✓		
Communication specialist with health professionals	✓		
University professor of nursing	✓	✓	
Research assistants with mental illness	✓	✓	✓
PhD candidate in nursing	✓		

*Authors of this paper

Close collaboration and consultation between the Advisory Board and Co-Research Team was undertaken at all key stages of development, with each draft of the IGMA taking account of their input and feedback. The IGMA was therefore developed and implemented through a participatory process with a local health organisation that ensured key stakeholders were able to review and provide comment on the final production of the IGMA. Some members of the Advisory Board were involved in the selection of the IGMA questions. The Advisory Board is also very actively involved in the planning of the small group learning schemes that are offered to GPs through the continuous medical training activities of the East-end Montreal local health authority, for them to be informed about the concept of diagnostic overshadowing [2] and to discuss ways to better integrate physical and mental health care in primary care. Advisory Board members are also important conveyors of the ongoing knowledge translation strategies.

### Study participants and nature of participation

SMI affects around 0.3–0.7 % of people at some point in their life [20]. Therefore, for a population of 100,000 inhabitants, approximately 500 persons could be affected by SMI at any time. The IUSMM Archives Department provided a list of 467 patients with SMI who live one the East-end Montreal sector covered by the local health and social services centre, according to their postal codes. From those who were contacted by phone by research assistants who were using that list, 146 patients agreed to participate and have completed the IGMA between September 2014 and January 2015. This 31.3 % participation rate is almost exactly what could have been expected, according to Mojtabai *et al.* for a study on a similar topic [21]. 95 of them were males (65.1 %) and 51 were females (34.9 %). Systematic reviews show that it is more common in men than women with a risk ratio of 1.4:1 [22]. The mean age is 52.7 years old with a standard deviation of 14.8 years.

Study participants are expected to meet their GPs (T2) to show them the results to the IGMA within 12 months after T1, and they receive a 50$ compensation for each T1 and T2. In effect, one objective of the larger study is to assess whether there will be a change in terms of health promotion and follow up trajectories by comparing the rates of avoidable admissions or procedures to other psychiatric patients of the East-end Montreal sector and to the general population of the province of Quebec after T2. This paper reports the results at T1, as collected with the IGMA that was used by study participants who were met in small groups of up to 5 participants each. Approximately half of these small groups were held at the IPPAR-IUSMM and the other half were held within specialized residential resources for participants with lesser autonomy. The patient research partners of the co-research team were following a script for the conduct of these small group learning meetings and they were assisting the study participants in completing the IGMA questionnaire. More than 95 % of study participants were using an electronic tablet for the fist time.

The IGMA was developed through a series of iterations and field notes were taken to observe and document the participatory R&D process. A psychiatrist (one of the investigators) and a GP (member of the Advisory Board) were asked to independently select among 150 questions that were gathered by a patient who is also a physician and research assistant, as per the above Table 1. She used her experience and went back to classic textbooks of medical education [23, 24] for this wide range of clinical issues and questions that it would ideally be recommended to cover for a comprehensive medical history of patients. From the 150 questions the psychiatrist and the GP independently identified those conditions that seemed to them to be the most important to be documented in an individual medical record of any patient. They selected about 50 questions each, of which 30 were common to both the psychiatrist and the GP. After discussion with the Advisory Board, 3 more questions were added. The 33-item electronic form produces an individualized profile of medical history which is printable on a single sheet to be shown by the patient at the time of the medical appointment with a GP. The items covered by the Australian Guidelines are also covered by the IGMA and the 33 questions of the IGMA can be answered on a binary scale (yes-no questions) [25]. Yes-no questions are typically used by medical staff when they ask patients to fill out questionnaires in waiting rooms.

For each of the IGMA questions, the patient research partner with medical training prepared a description of the covered symptom or sign. The 33 descriptions were based on official medical literature and approved by the abovementioned practicing GP who took part in the R&D process of the IGMA. They were gathered in a 33 pages paper document with a selection of recommended medical websites for the reader to learn more about a particular condition and on how to possibly prevent this condition with improved health literacy and behavioural change. Two other mental health service users and patient partners were asked to carefully read these descriptions along with the questions. They thus encapsulated 33 short videos with their lay language, and their colleague with medical training was there all the time to ensure that every important aspect was appropriately covered. The IGMA was presented to the Advisory Board members who approved the content and the approach, including the videos, and study participants began to use the IGMA (T1). In a future iteration of the IGMA, we plan to provide additional aids like graphs or other interactive aids to help the viewers better understand the questions.

In the next phase of the study, we will measure outcomes from the use of the tool by comparing hospital separations including diagnoses and procedure codes, and physician billings including diagnoses and procedures. Results for the semi-experimental group of patients with SMI will be compared to that of the other psychiatric patients of the East-end Montreal sector (Control 1), the other SMI patients of Quebec (Control 2), and Quebec’s general population (Control 3). Data sources are the administrative data of the *Régie de l’assurance maladie du Québec* (RAMQ) for these 4 groups. Matched controls (Control 3) will be identified within the RAMQ routine data sources as patients without SMI, matched for physical co-morbidities in the previous year, using the Charlson-Deyo comorbidity index, a validated method of classifying comorbidity to predict short and long-term mortality from medical records [26, 27]. To add to the power of the study we aim to identify at least five controls to every one case [28], and matched controls will be identified within the RAMQ as live individuals matched on age (to the nearest year) and gender who were registered within the RAMQ for the non-affective diagnoses codes at least 1 year prior to matched cases of the intervention group for Controls 1 and 2, and for physical co-morbidities for Control 3.

## Results

Participants were offered coffee and muffins, and then viewed each video before answering the corresponding specific question on the touch screen of an electronic tablet. On average, it took 90 min for participants to view all the 33 videos and complete the questionnaire. After completion, they were given the paper document, including their individual answers listed on a single sheet that resembles Table 2, which presents the results in percentages of participants who answered “yes” to the IGMA questionnaire at T1 (*n* = 146 patients who all participated in small group sessions).

**Table 2:** Results to the Interactive Guide for Medical Appointments (IGMA) at T1 (*n* = 146)

#	Question	Yes
1	Have you ever been treated for a heart disease?	17.6 %
2	Have you ever been treated for high blood pressure?	28.9 %
3	Have you ever been treated for diabetes?	35.9 %
4	Have you ever been treated for chronic bronchitis or emphysema?	17.6 %
5	Have you ever been treated for asthma?	18.3 %
6	Have you ever been treated for cancer?	11.3 %
7	Have you ever been treated for thyroid malfunction?	14.8 %
8	Have you ever been treated for chronic pain?	23.9 %
9	Has one of your relatives been victim of a cerebrovascular accident (stroke), of a heart disease or of cancer?	62.7 %
10	Do you suffer from allergies?	34.5 %
11	Have you consulted with the emergency or walk-in clinic during the past year?	35.9 %
12	Have you lost weight unintentionally over the last 6 to 12 months?	14.8 %
13	Do you have pain that wakes you up at night?	20.4 %
14	Do you have frequent or severe headaches limiting your activities?	18.3 %
15	Have you fainted recently?	3.5 %
16	Do you have any abnormal movements or tremors?	35.9 %
17	Do you frequently feel out of breath?	26.8 %
18	You spit blood when coughing?	0.7 %
19	Have you ever had blood in your stool or black stools?	13.4 %
20	Have you noticed blood in your urine?	3.5 %
21	Have you noticed an increase in the frequency with which you urinate or an increased volume of your urine?	26.1 %
22	Do you have any unusual discharge from the vagina or the penis?	4.9 %
23	Have you noticed a change in the appearance of your moles?	5.6 %
24	Have you ever been tested for colon cancer over the past 2 years? (50 to 74 years old)	13.4 %
25	Have you had a gynaecological examination including a screening test for cervical cancer (PAP test) in the last 3 years?	37.3 %*
26	Have you had a mammogram screening for breast cancer (women aged 50 to 69) in the last 2 years?	33.4 %*
27	Have you been screened for sexually or blood transmitted infections?	12.0 %
28	Do you smoke?	56.3 %
29	Have you ever thought you should cut down on your drinking or have you ever been criticized by people around you because of your drinking?	4.2 %
30	Do you use drugs (marijuana, heroin, cocaine, LSD, ecstasy, crystal meth, etc.) or psychotropic substances without prescription such as stimulants (e.g. *Ritalin*), painkillers (e.g. *Fentanyl*), sedatives (e.g. *Valium*), etc.	7.7 %
31	Do you do 2.5 h of physical activity of moderate to high intensity in a week?	23.2 %
32	Do you eat at least seven servings of fruits and vegetables per day?	20.4 %
33	Do you brush your teeth every day?	47.2 %

*Women only

Thus, with regards to participating patients, data collection has been completed for T1. All participating patients effectively viewed the videos and successfully completed the questionnaire. They left the small group learning sessions with a paper copy of the IGMA and questionnaire results for sharing with their GPs (73 have already done so as of May 31^st^ 2015). Field notes indicate that these small group learning schemes represent an acceptable means of gathering patients with SMI for them to discuss and be more aware of their own physical health condition. Among the limitations, though, is that we cannot yet conclude to the effectiveness of the tool in terms of care pathways and health outcomes for study participants compared to other psychiatric patients of the East-end Montreal territory, nor to the general population of the province of Quebec because their GPs have not yet been met. The Direction of Family Medicine of the East-end Montreal local health authority has agreed to facilitate and coordinate the participation of the GPs, for them to be met, too, in small group learning sessions to discuss ways to better integrate physical and mental health care in primary care. Other potential barriers to effective implementation are the perceptions of incompetence, of dangerousness, and of permanent impairment that health care providers, like the public in general, can have towards patients with SMI [29]. The active and visible participation of patients in the small group sessions among health care providers should help to generate a more positive image and to strengthen GP’s confidence in engaging themselves in health promotion dialogue with SMI patients.

To improve their health literacy, participating patients were exposed to the 33 videos and each time they were suggested reliable medical websites for further exploration in the paper document with which they were leaving the small group sessions. Their perception of their own physical condition was documented by answering the 33 questions. They were given their own individual results (the last page of the IGMA paper document to be shown to the health care provider) and Table 2 presents the aggregated results (*n* = 146).

These results can be combined in multiple ways and be cumulative in their effects. Some patients can have multiple chronic diseases, which are more or less incapacitating. For others, a single one can have as much consequences as a combination of several “milder” conditions. Combinations can vary in terms of number and/or intensity, and nature of the diseases. Emphysema and asthma are reported by about 18 % of participants, so many frequently feel out of breath (27 %). About 36 % have abnormal movements or tremor, which may be side effects of psychotropic medication. Each of the single results would deserve a detailed analysis, for example in comparing the 35.9 % prevalence of diabetes among study participants to 8.3 % for the population of Quebec [30].

The results could also be presented in combination. An example of complex combination, for instance, is morbidity due to oral disease. As shown in Table 2, not even half of study participants self-reported that they do brush their teeth. More than 55 % still smoke (56.3 %). These are changeable lifestyle habits. Better management of oral health is possible through the treatment of oral diseases and prevention but among the most common somatic disturbances observed in SMI patients is their poor oral health [31]. Prevention implies well-educated hygiene, rational use of complementary devices, the acquisition of manual dexterity and regular reactivation of guidelines and recommendations. In effect, to limit the development of dental plaque, brushing is an effective mechanical action that prevents gingivitis. The preventable deterioration of oral health and its impact on vital functions, especially as it relates to nutrition, may affect the overall health and metabolic conditions of individuals. Improved overall quality of oral health would decrease the need for curative treatment, which are often invasive (use of “conscious sedation” or general anaesthesia).

Also to note is that these results are self-reported, they do not replace a medical report. It is thus very much possible that those 47.2 % (brushing their teeth) and 56.3 % (smoking) are embellished numbers due to social desirability. Another social dimension and outcome is that the oral cavity plays an important role in the aesthetic appearance. Tooth loss symbolizes vital physical decline with all its psychological consequences, for example in terms of self-confidence in seeking and maintaining healthier life habits, and in terms of communication skills and presentation style. This is also probably true with many other conditions. What the next section discusses are not the numbers per se. Rather, it is the participatory R&D process and use of the IGMA as an interactive tool for data collection and social interaction.

## Discussion

In the Region of Montreal, when individuals are looking for a family doctor in primary care, they need to fill out an electronic form that is provided by the local health authority on its website. The IGMA questionnaire covers these basic questions, with the addition of a number of questions that were based on 3 expert opinions. Although these Montreal-based experts do have a professional expertise about SMI patients’ particular needs of supplemental assessment with regards to their physical condition, this is a limitation compared to systematic approaches like systematic literature reviews, clinical guidelines, or expert consensus.

In recent years, mobile applications and such electronic forms have become part of daily life for many, and they have also entered the medical field [32]. For example, such applications can be used by patients who would like to quit smoking [33], to lose weight [34], or to assess anxiety [35]. Some populations, however, remain significantly disadvantaged in terms of access to such technologies, as is the case with the vast majority of participants in this study who had never handled an electronic tablet or completed an electronic form before using the IGMA. They nevertheless were able to do so, though with some support from the patient research partners. As health institutions are more and more referring the population they deserve to their websites for information and registration, they should be aware of groups with lower Internet access when planning to offer consumer health information technology. Responding to the needs of such socioeconomically disadvantaged groups is challenging for family physicians and primary care teams [36, 37]. This is an important issue in terms of equity of access for under deserved groups who are at risk of being technologically left behind [38].

There were a number of challenges that required special assistance during the process. For example, at times, it was difficult for some participants to stay focused on the task. The team of 2 peer research assistants ensured that there was minimal distraction during the small group sessions. Also, for some transportation was an obstacle. In such circumstances, the team of peer research assistants went in specialized housing resources with five electronic tablets that were connected to the internet. The leadership and input of patient research partners of the IPPAR have been useful in making the data collection procedure a positive, empowering and genuinely personalized experience that many of them might want to repeat in the future. The fact that study participants were met by some of their peers helped avoid inequalities in dialogue, as the study participants’ full participation was not inhibited by unequal power relationships [10]. Interactivity, here, may be more about this equalitarian type of interaction than about multimedia and touch screen technology.

The active participation of patients is a key component of patient-centred research for stimulating the translation of scientific and technological innovations into benefits for patients [39]. The patient-centeredness of this participatory research was characterised by an enhanced level of patient commitment to this R&D process of the IGMA and its use. Patient-centeredness can contribute to better integration, continuity and fluidity among various stakeholder groups [40] and research is increasingly demonstrating the usefulness of including patients and considering their preferences in health research [41, 42]. Innovation and investments in patient centred research have become highly prioritized in the United States [43], the United Kingdom [44] and Europe [45], and Australia [46]. This project is part of that trend, with clinicians and patients working in close partnership to improve healthcare and challenge deeply ingrained practices and behaviours [47]. Partnering with patients, their families, carers, advocacy groups, and the public is more and more seen as an ethical imperative that is essential to improving the quality, safety, cost effectiveness, and sustainability of healthcare. Especially in the field of mental health, the practice of patient partnership can be seen as the culmination of decades of claims from advocacy groups demanding equality, equity, and full citizenship for all [48].

Still often, new mobile applications are designed to be used by professionals [49], and with a predominately individual-based approach [50]. However, “individualized” is not synonymous with “personalized”. A system that is accessible is made up of health professionals who are accessible, but beyond opening hours, patients from lower social classes are often disadvantaged because of the doctor’s misperception of their desire and need for information and their ability to take part in the care process [11]. A tool like the IGMA can help patients to better communicate with the GPs [51].

This study illustrates the relevance of participatory action research in addressing an important public health topic. People with mental illness and family members were included in several ways. One was a co-investigator, 2 others formed part of the Advisory Board and 2 more were employed as peer research assistants. Then, a young woman and a young man with SMI made the videos, for a total of 7. Work in the mental health field is attractive to many consumers, as it can offer consumers the chance to socialize and help their peers [52]. Hiring patient research partners who otherwise would be unemployed [53] is yet another relevant way to tackle inequity.

In their systematic review to assess the interformat reliability of self-report symptom scales used in digital or paper format, Alfonsson, Maathz and Hursti [54] have concluded that when digital versions of self-report symptom scales are compared to pen and paper versions, most scales show high interformat reliability. For our study, participating patients were offered to complete the IGMA in its electronic or traditional paper format interchangeably. The focus was on the dynamic that took place among participants, with some of them completing the electronic questionnaire and only very few of them preferred to complete the paper-pen questionnaire. In either way, they all had to view the short videos before answering each question and one of the reasons why the overall process lasted up to 90 min was that participants tended to discuss among themselves in order to exchange some tips, for example, as how to better control this or that particular condition. In effect, each time the content of the videos was describing a common medical condition in a lay language for participants to be able to eventually recognize it. Participants were explained that the narrators in the videos were also people living with SMI and this information probably gave them confidence in engaging themselves, too, in the discussion. Finally, through this study, participating patients have experienced information and communication technologies by filling out an electronic questionnaire, a premiere for several of them. As explained in the IGMA that is shown to the health care providers, their answers to these 33 questions do not replace a medical diagnosis or record. Nevertheless, so far the use of the IGMA has been shown to be useful in raising awareness about the diagnostic overshadowing phenomenon because health care providers can see, so to say “at a glance”, that some of their SMI patients have a long history of combined physical conditions.

## Conclusion

The main objective of this paper was to explore and confirm the feasibility and acceptability of patient partnership for developing an interactive guide to improve access to primary care providers for chronic diseases management and health promotion among patients with severe mental illnesses. Difficult communication and social distance have been identified in the literature as barriers to equitable access to primary care providers and this access can be improved through the use of a tool like the IGMA and the interactivity and collective social support that its use generates among small groups of patients.
